# Coercion rates in different mental health care models: flexible assertive community treatment vs care as usual

**DOI:** 10.1192/j.eurpsy.2023.106

**Published:** 2023-07-19

**Authors:** A. Tomcuk, RECOVER E Montenegro Research Team

**Affiliations:** Special Psychiatric Hospital, Kotor, Montenegro

## Abstract

**Abstract: Introduction:**

In 2018, within the Horizon 2020 program, RECOVER-E project activities were initiated in Montenegro. During the years 2019 and 2020 Community mental health team (CMHT) within the Special Psychiatric Hospital Kotor was established. This team became responsible for management of treatment of a group of users with severe mental health illnesses, based on the principles of „Flexible Assertive Community Treatment (FACT – A Dutch model). Objectives: The main objective of this research was to establish whether there were substantial differences regarding the use of seclusions, restraints and forced medication during the hospital readmissions in the group of patients treated by the CMHT, compared to usual mental health care in Montenegro.

**Methods:**

A sample of 202 users of mental health services from Kotor and surrounding municipalities were recruited. Patients were randomized into two similar-sized groups - intervention group, whose treatment was managed by the multidisciplinary CMHT, and control group where treatment as usual was continued (outpatient treatment without field work and hospital readmissions during the psychotic relapses).

To estimate and follow-up the frequency of application of coercive measures in this research, hospital documentation was used.

**Results:**

Patients in the intervention group had statistically significant less coercive intervention (such are mechanical restraining, seclusions, isolations and forced medication) during the study. There were no significant differences in the number of hospital days and readmission rates.

**Conclusions:**

This study showed that CMHT care could reduce some of the coercive measures during the treatment of severe mental illnesses.

**Disclosure of Interest:**

None Declared

